# Assessment of new public management in health care: the French case

**DOI:** 10.1186/1478-4505-12-57

**Published:** 2014-10-06

**Authors:** Daniel Simonet

**Affiliations:** American University of Sharjah, Sharjah, PO Box 26666, United Arab Emirates

**Keywords:** Centralization, France, Hospitals, New public management

## Abstract

The French health care system embraced New Public Management (NPM) selectively, and crafted their own version of NPM using Diagnostic-Related-Group accounting to re-centralize the health care system. Other organizational changes include the adoption of quasi-markets, public private partnerships, and pay-for-performance schemes for General Practitioners. There is little evidence that these improved the performance of the system. Misrepresentation has remained high. With the 2009 Hospital, Patients, Health and Territories Act physician participation in hospital governance receded. Decision-making powers and health units were re-concentrated to instill greater national coherence into the health system.

## Commentary

### The path toward new public management

European economic integration with policies dictating that the achievement of supranational objectives prevails over national and regional policies, the adoption of a monetary convergence criteria (to illustrate the point, national budget deficits are capped at 3%), and the perception that Keynesianism failed in the 1980s, all prompted French public policy makers to adopt a new market-based paradigm to drive economic policy [1]. Under the impetus of top-politicians, consulting firms (the transfer of corporate management recipes to the public sector provided them with a steady source of income) [2], and liberal think-tanks [3, 4], a new generation of civil servants from the French National School of Public Administration and the “Grandes ecoles” (i.e., the French equivalent of US Ivy League universities) – a “*programmatic elite*” [5] – who, like the public, wanted a delivering government [4], embraced New Public Management (NPM) [6] in a bid to improve public services delivery. Private hospitals and hospital bureaucrats who view these reforms as legal (they were voted in Parliament) and rational because they respond to a need for a more efficient allocation of resources, also supported the NPM. In contrast, the operating core (the medical profession) usually resisted NPM reforms, albeit with mixed success due to the rising division of physician trade unions [7]. Local notables, such as mayors and their constituency, who oppose the closure of smaller hospitals in rural areas, also questioned this neo-liberal progressivism. As an instrumental value, ‘efficiency’ may serve competing interests and mean different things to different groups. From the government perspective, efficiency implies budget discipline and an emphasis on lower costs. For private operators, it implies market share expansion. For patients, it means shorter waiting times. That ambiguity allowed NPM to permeate the health system in a politically correct manner.

EU integration was also a factor. Budget deficits, taxation levels, and the share of health expenditures in the GDP – rather than quality indicators such as life expectancy and infant mortality – became essential tools in comparing country achievements. Key NPM concepts, such as the use of market forces to serve public purposes [8], activity-based payments, management by objectives, quasi-contracts (as in the British NHS [9]), and outsourcing, made their way into French national policy [10]. Emphasis was placed on benchmarking [11] and performance evaluation [12, 13]. Programs were broken down into missions subjected to performance indicators or “*public management by numbers*” [14].

Scott et al. [15] see healthcare as a rapidly changing environment with defined timeframes; the era of professional dominance (1945–1965) with an emphasis on quality; the era of federal involvement with a focus on greater access via Medicare and Medicare program (1966–1982); and a managerial era with a reliance on market mechanisms and entrepreneurship. Likewise, France emphasized the NPM entrepreneurial hospital model over the ‘traditional’ compassionate (e.g., care provision to the destitute and the vulnerable) and professional (e.g., physician training) hospital models [16, 17].

### Assessment of overseas NPM experiments

Earlier assessments of NPM, notably in Commonwealth nations, point to outcomes below expectations. “*The commoditization of deregulated health systems makes them very inefficient and very expensive and it exacerbates inequalities and leads to a deterioration of the quality of care*”*,* the World Health Organization (WHO) concluded in its 2008 report on health. Though the way in which the “*NHS operates was changed irrevocably by the quasi-market reforms*”, there were relatively “*little measurable changes that could be related unequivocally to the core mechanisms of the quasi-market*” [18]. A culture of ‘glossification’ or investment designed to impress rather than to provide better outcomes permeated the NHS [19]. For some [20–22], competition led to better results. For others [23–25], competition, specifically price competition, had no impact on health outcomes. In Australia (and also Italy), the largest outsourcing contract in public health of the 1990s was unsuccessful [26, 27] since major contractors failed to deliver [28, 29]. A merry-go-round of outsourcing and reintegration followed the outcry of the medical profession and the media. Market orientation had a negligible effect on performance judgment of local managers or the Audit Commission [30]. New Zealand, an NPM index case, abandoned its quasi-market structure [31] after it failed to tackle cross-government problems, notably in the areas of health and pediatric wellbeing [32], and has moved toward national coordination and centralization of some planning and service delivery functions [33]. In Australia, there “*appears to be no particular benefit to service users of quasi-market reforms, particularly in policy contexts where service delivery systems are historically under-funded*” [34]. Moreover, market transaction costs probably surpass potential savings that quasi-markets may generate. In other non-Commonwealth nations, such as Italy, the government of Lombardy “*deliberately sacrificed competition in order to control health expenditures*” [35]. A comprehensive review of European Public-Private Partnerships revealed mixed results [36].

Other ‘traditional’ NPM tools [37], such as general practitioner (GP) pay-for-performance schemes, too had a limited impact [38] or no impact at all [39] in foreign exemplars. The US [40, 41] and UK governments struggled to pay for performance or better outcomes. Vogel [42] suggests that “*the often assumed superiority of managerial over bureaucratic control is constructed through discourse rather than being a law-like regularity*”. Emphasis on performance evaluation appears time-consuming, confusing (e.g., the use of management instruments to improve the performance of public services does not always simplify the public management exercise) [43], and expensive [44]. Moreover, it neither prevented poor quality of some care providers [45], nor led to a more efficient or accountable management at the local level [45]. In foreign exemplars [46, 47], audits, constant monitoring, and performance measurement have spillover-effects such as ambiguous responsibility and increased complexity. As accountability is increasingly shared and thus dispersed among multiple stakeholders, clarity is lacking [48] and its relationship with performance is increasingly contested [49]. Often, evaluation reflects differing perceptions of insurance and performance rather than actual performance improvement [50]; hence, there is a need to refine accountability for a greater public good [51]. This, however, is harder to achieve in health care than in other public areas due to cultural differences between physicians, administrators, and politicians. The intersection of professional culture, rules, and patient needs creates instances of both rule abidance and deviation, all of which affect quality [52]; therefore, the performance-enhancing (or -obstructing) effects of targets and rankings will vary according to the culture in which they operate and the level of unorthodoxy it tolerates [14].

During the 1990s, decentralization of decisions from the central government (the Ministry of Health) to regions and locally-elected officials was seen as a way to open governance to a wider participation of the public and civil society [53]. In France, for instance, the city mayor was the chairman of the hospital supervisory board. Decentralization, however, was not necessarily more democratic [54] and proved expensive, as seen by an increase of 124% and 64% of the debts of regions and counties (or *Departement*) in a decade.^a^ Moreover, it led to rising discrepancies in accessing care [55] and regional policies’ efficacy was questionable [56].

### French selective adoption of NPM

Health systems and their policies are complex social phenomena that are not only shaped by human action, rather than naturally occurring [57], but are also constrained by historical, social, and institutional arrangements. Far from constituting a single uniform paradigm, NPM is a doctrinal makeshift or ‘doctrinal puzzle’ [58] that is also influenced by national characteristics such as centralization in France. Decentralization is a relatively recent phenomenon that dates back to the 1980s and contrast with its Napoleonic tradition of centralization [56].

We undertook a review of current studies to evaluate NPM impact on the French Health Care system. The paper argues that France strengthened the vertical chain of authority to reinforce the central government’s role in the definition of a national health policy. In the mid-1990s, a ‘wave’ of agencification occurred in the areas of public health. The Juppé reforms (1996) created the Regional Hospital Agencies (RHAs) that took over some, but not all, responsibilities from the Ministry of Health, such as the County Bureau of Social and Sanitary Affairs or *Direction Départementale des Affaires Sanitaires et Sociales* to determine health priorities with the input of the Regional Health Conferences and the Regional Committees on Health or *Comite Regionaux d’Organisation de Santé*. RHAs introduced novel management practices [57] such as multi-years performance contracts with care providers. On top of that were the National Accreditation and Evaluation Agency and a High Authority on Health. In recent years, however, France rejected the decision on decentralization of health care and, more generally, the NPM-endorsed disaggregation of health agencies that were traditionally advocated in Anglo-Saxon countries [58–60]. The 2009 Hospital, Patients, Health, and Territories (HPST) Act enforced a re-concentration of all health responsibilities – not just the hospital prerogatives of the earlier RHA – into a Regional Health Agency. Moreover, the smaller regulatory and service delivery agencies (the Regional Public Health Groups or *Groupements Régionaux de Santé Publique*, the Regional Health committees or *Missions Régionales de Santé*, and the Regional Sickness Funds) were regrouped into the Regional Health Agencies. France adopted contractual agreements for its care providers and focused on outcome-oriented rather than input-based budgets. However, despite reiterated calls for greater accountability and transparency [61] and a movement towards an ‘audit society’ [62], misrepresentation has remained high [63]. Finally, French NPM reforms restricted democratic planning and led to a re-concentration of health organizations to achieve greater national coherence.

### Diagnostic related groups (DRGs) and the quest for central government control

Originally derived from the 30-year-old Program of Medicalization of Information Systems or *Programme de Médicalisation des Systèmes d’Information*, activity-based payment or *Tarification a l’Activité* is the French version of the US Diagnostic-Related Groups (DRG) (i.e., a group of patients with medically-related and economically-comparable diagnosis), as defined by Fetter [64]. Unlike the former fixed global budget based on regional demographics, such as population size and age covering all hospital expenditures, DRGs, referred to as Homogenous Groups of Patients (‘*Groupes Homogenes de Malades*’) in France are pre-determined reimbursement rates that reflect services that are actually delivered. DRGs are a good proxy for the broader concept of NPM, because DRGs draw on two of its basic principles: firstly, a split between financing, primarily from the government, and care provision by hospitals; secondly, incentivisation and competition for patients [60, 65], as care providers’ income is directly related to patient volume [66]. This uniform DRG rate schedule for all hospitals nationwide replaced the former hospital financing model that depended on patient length of stay, local demographic factors such as population size, and the hospital bargaining power during fee negotiation with the central government. With care providers’ funding currently depending almost solely on DRGs (their penetration rate increased from 10% in 2004 to 100% in 2011 for French public hospitals, the highest in Europe) [67] and other supplementary payments, for instance for research and training, hospitals are rewarded, or punished, for their economizing behavior. Poorly-performing units, such as hospital wards with higher costs or fewer patients, must return to profitability or close, as observed among other public organizations that embraced similar competitive payment mechanisms [68]. The French Social Security offers incentives to care providers such as a repayment of a fraction of the savings achieved, which can be used to acquire new equipment or to improve staff working conditions.

Due to the NPM’s greater focus on accounting and budgeting issues, the geographic, economic and administrative (i.e., the re-centralization or the need to ‘reassert the center’) agenda of DRGs was largely ignored. These, however, had the potential to prevent regional disparities, a longstanding characteristic of the French health care system [69, 70]. The rise of the regional authority in the 1990s [71] caused ‘inefficiencies’ such as a ‘Balkanization’ of the health system, as exemplified by rising health disparities across regions [72], which is the burgeoning of local health organizations (regional health forums and conferences) with each having their very own policy agenda that sometimes contradict the national health plan that DRGs intend to remedy. Hospitals were traditionally run by municipalities; often, the city mayor doubled up as the director at the hospital board. From an administrative perspective, DRGs prompt hospitals to achieve centrally, rather than regionally-defined, cost targets, which, in theory, will restore the authority of the central government over regions [4].

From an economic perspective, DRGs would adjust hospital resources to actual clinical activity, thus preventing the inflationary effect of the former global budgeting and *per diem* payments. Moreover, these mechanisms were poorly regulated, complex, and unfair since budget depended on historic trends [73] but also on the provider’s ability to negotiate with the Social Security [74]. By gathering data on hospital costs across the country, thereby enabling a comparison of their ‘productivity’, DRGs would induce a nationwide standardization of care provision (DRG payments are identical for all care providers across the country) and, in theory, align medical practice with that of the most efficient providers, as in the NPM neo-Taylorist model [17].

From a political perspective, however, this re-integration questions the NPM-driven neo-liberal agenda. Centralization is a general thrust in the French political system that intends to restore the power of top-level bureaucrats who lost their prerogatives during the decentralization era of the 1980s and 1990s. The center (i.e., the Ministry of Health) developed the DRG scale as a tool to unify payments across regions so that hospital compensation no longer depends on past historic trends and on the bargaining power of local notables. Its intent was to de-politicize the management of regions [75] and to restore a fiscal discipline that was lacking among local politicians.

### The rise of quasi-markets, GP pay-for-performance schemes, and public-private partnerships

As in British quasi-markets (markets are ‘quasi’ rather than ‘pure’ since players are not necessarily competing for a profit, nor are they necessarily private, some are state-owned) [10], the French state is no longer a care provider (the term ‘public hospital’ has disappeared from the official government nomenclature in the aftermath of the 2009 HPST Act), but a financier of health services [76] that stewards or ‘steers’ the production of health services rather than produce or ‘row’ these services directly [77]. Contract-based management or *Contrats d'Objectifs et de Moyens* between Regional Health Agencies (*Agence Regional de Santé*) and hospitals lays the foundation of these quasi-markets, defining financing grants and activity targets for care providers, including patient volume, as in the NPM-endorsed corporate Management by Objective and Results approach [78]. France, however, did not go as far as the UK when it comes to quasi-market implementation because of stronger counter powers such as the operating core, e.g., the medical profession. In theory, hospitals and out-of-hospital care providers, public or private [79], compete for Social Security funding under the supervision of the Regional Health Agencies. In practice, however, activity-based payments are not determined by the market; hospitals are not permitted to set fees for their services – these are decided and administered by the central government based on a sample of participating hospitals [80]. Since prices are regulated, one can talk about fictitious competition [81]. Moreover, hospitals receive supplementary payments other than DRGs to fulfill some of their public service missions, including physician residency programs that public, rather than private, hospitals provide [82], or to pay for experimental treatment, with both payment types providing opportunities for cost-shifting. Moreover, prices alone are not enough to drive policy implementation, as care providers and patients do not spontaneously behave as price takers; hence, a GP pay-for-performance scheme or Contract for Improving Individual Practice (*Contrats d’Amélioration des Pratiques Individuelles*) rewards solo physicians with premiums of up to €5,000 for providing preventive services and for efficient prescribing in the treatment of chronic diseases. Three months after its introduction in 2009, more than 10% of GPs joined the scheme [83]. The 2007 and the 2012 National Hospital Plans recommended Public-Private Partnerships (PPP) to delegate certain services that are too expensive for a single public operator such as the construction and maintenance of hospitals. The private operator collects a lease over 30 years; the hospital will be handed over to the public sector after that period. PPPs have been rising fast (77 were adopted in 2011, 36 in 2010) under the Sarkozy presidency, with France pouring more money into PPPs (€3.5 billion in 2008 according to Constitutional Council) than the UK (€3.11 billion according to the UK Treasury, 2011), an NPM index-case. PPPs in health care accounted for a €6 billion investment in 2011 out of €90 billion in total public investment [84]. However, that number has experienced a steady decline with only 36 PPP from January till October 2012 and 17 from January till October 2013.^b^

### A critical assessment

Evaluation of French NPM reforms on health services’ efficiency point to outcomes below expectations. Despite a long tradition of cost/benefit analysis among French bureaucrats [85] via the adoption of a rational model of public budgeting in 1970 (i.e., ‘Rationalization des Choix Budgetaires’), assessments based on simple health indicators, such as mortality rates and other measurable health outcomes, including hospital readmission rates, length of stay, or quality of care, are not routinely available in France [86]. Therefore, from a medical perspective (changes in the quality of care, improved health outcomes), the impact of NPM is difficult to ascertain. Instead of improving or simplifying government execution, it appears that constant monitoring created additional risks [87] and costs [88] that remain largely un-quantified, such as accounting (French hospitals hire ‘DRG-coders’ when they should hire physicians) and social costs. Moisdon and Tonneau [80] observe that “*adopting tariff is to accept an implicit planning that benefits the most efficient care providers, leads to a supplier concentration and a withdrawal from certain areas*”. The medically underserved areas (rural areas suffers from a longstanding shortage of GPs) fare worse under NPM, as more public hospitals close in geographic areas that do not support a sufficient patient pool [89]. On top of this adds the closure of medical services that are too expensive to run such as cardiology, nephrology and emergency services in city-centers [16]. Discrepancies in hospital payments, the driving force behind DRG adoption [74], have not disappeared, but were merely shifted from the central government to the patient who carries a higher share of the cost. Despite a nationwide uniform DRG scale, daily fees, which are set by the hospital director, approved by the RHA, and paid for by the patient and his/her supplementary insurance, varies significantly. In a survey on medical cost in city-based hospitals, procedure costs ranged from €360 euros to €2,230 for a similar medical condition with an average cost of €817. Therefore, fees still do not reflect care intensity, but constitute an adjustment mechanism to balance the hospital budget [90].

The public/private sector gap is rising. Private hospitals focus on the most profitable DRGs (including ambulatory care, elective surgery, and maternity care) [73], leaving services with poor returns, for instance, organ transplantation or emergency services, which are more expensive to run, or welfare services (often, emergency rooms double up as shelters for the homeless), to the public sector. Compounding factors are patient selection, which were described early on in foreign exemplars [91, 92]. Therefore, one cannot yet talk about ‘hybridization’ or the concomitant development of professionalization [93] and the streamlining of the public sector for a balanced budget. The level of debt of public hospitals even trebled between 2003 and 2011 [94]. Thus, regarding the frugal use of public funds, as endorsed by NPM, outcomes are wanting.

As for PPPs, particularly for the construction of hospitals, outcomes are clearly negative for the tax payer. Early PPP evaluations point to inadequacies. According to the regional General Accounting Office [95] that reviewed the largest hospital construction site in the city of Evry, a simulation for a 30 year public loan of €344 million cost a total of €757 million compared to the €1.2 billion that were actually paid to the private operator Eiffage. Unlike a private operator that contracts loans at higher rates or invests its own funds with an expected return on investment of 10% to 15%, public operators benefit from lower financing costs since they can borrow at preferential rates. These failures prompted the French government to adopt more stringent regulations to ensure that PPPs do not bankrupt municipalities. According to Decree No. 2012–1093, voted on September, 27, 2012, municipalities must evaluate the impact of every prospective PPP on their finances and credit rating before approving it. With municipalities being unable to manage their budget and mayors embarking on expensive investment with intergenerational implications, the government had to set up a fund (2014 Finance Orientation Law) with a yearly appropriation of €100 million for the next 15 years to bail out local governments that were unable to repay the debts that PPP incurred. Current PPPs in Caen, Annemasse, and Saint Nazaire will help determine whether PPPs are economically-viable strategies, but with the arrival of a new Prime Minister Manuel Valls who is known to be hostile to these arrangements, PPPs are likely to be delayed further.

### Can NPM prevent misrepresentation?

The impact of NPM on fraud and abuse is not a mainstream issue in the NPM debate. On the one hand, it is argued that competition rather than cooperation, greater transparency, and physician liability, via output budgeting, can help prevent fraud, or at least detect it [96]. On the other, George Frederickson asserts that “*in democratic settings, government agencies and their officials in bureaucratic hierarchies are more ethical than self-interested individuals or firms in competitive markets*” ([97], p. 300). According to Grunow [98], the quest for efficiency, a flatter hierarchy which reduces the scope and intensity of control, and the decentralization of the decision-making process that entrusts more people with power over funding, have undermined other equally important public values such as integrity and public interest. Grunow [98] observed a steep increase of up to 700% of corruption cases in the German public administration that coincides with the introduction of NPM in the mid-1990s. In theory, DRGs enhance transparency and accountability; in practice, however, their inherent characteristics make them prone to ‘gaming’ the system. Physicians can adopt new opportunistic behaviors by manipulating DRGs, for instance, by ‘up-coding’ diseases. DRG codes with higher compensation rates can be attributed to the same disease (also known as DRG ‘creep’). Hospitals reduce that risk by hiring DRG-coders which increase costs and therefore contradicts the NPM cost-cutting agenda. Because of that ‘decoupling’ [99] or “*disconnects between formal structures and what individuals actually do in the course of action…*”, ethics and integrity may not necessarily be higher. France has the second highest estimated insurance and health care fraud losses (i.e., €10.576 billion) among 27 EU nations [63]. Exposure of fund mismanagement did not enhance accountability, and though it has not yet demobilized and bred resignation, public indignation remains low [100]. French citizens are less concerned with health care fraud than they are with others types of fraud such as corporate and bank fraud, they are more preoccupied by other competing issues such as unemployment [101], and remain divided over other societal issues such as immigration and same sex marriage [102]. Therefore, the government has little incentive to tackle the fraud issue more effectively.

### The democratic recess

Centralized – rather than regional – government intervention has been rising under NPM. Top-down decision-making by the Ministry of Health, regarding personnel appointment and the definition of a DRG scale by the Ministry of Health for all hospitals across the country, supplants the NPM-endorsed decentralized bottom-up policy making [53]. Under the 2009 HPST Act, the Ministry of Health appoints hospital managers (they used to be elected by their peers rather than appointed) and regional health agency managers. These can also be hired from the private sector (being a civil servant is no longer a pre-requisite to manage a health agency), and were given extended power and incentives (for instance, they can be fired if they do not meet centrally-defined targets) to achieve their objectives. By strengthening the power of centrally-appointed hospital directors and regional health agency managers at the expense of other traditional stakeholders, including city mayors, health care professionals, and trade-unions (to illustrate, public hospitals are often the largest employers in smaller cities) [103], the 2009 HPST Act intended to bring coherence to the national health system and reduce conflicts at the hospital strategic apex level. Sarkozy demanded “*a real boss at the hospital*” [104].

The hospital governance reform contained in the 2009 HPST Act ended regionalization [57] and the ‘democratic’ strategic planning of hospitals. The hospital board of directors was replaced by a supervisory board and the executive board by a managing board, with both having reduced power (art L. 6141–1 of Public Health Law). Therefore, the NPM-endorsed transparency is not always associated with a greater participation of the operating core [105]. Contracts with incentives are expected to yield better results in a single vertical hierarchical structure with clear power lines running from the Ministry of Health and its Office of Hospitalization and Organization of Care (*Direction de l’Hospitalisation et de l’Organisation des Soins*) to the hospital via the Regional Health Agency.

### Achieving national coherence via re-concentration and re-centralization of health care decisions

The 2009 HPST Act also merged regional institutions (for instance, Regional Association of Sickness Funds or *Union Régionale des Caisses d’Assurance Maladie*; Regional Directorship for Sanitary and Social Affairs or *Direction Régionale des Affaires Sanitaires et Sociales*) into a single ‘one-stop shop’, the Regional Health Agency, that complies with a nationally-defined health expenditure target. This re-concentration which extended to hospitals [106] intend to increase the capacity to govern that was lacking among early NPM adopters. Moreover, the widespread adoption of information technology [107], such as electronic medical records and accounting systems in the new digital-era [108], will ease the collection of health data, reduce transaction costs [109], eliminate the information asymmetry that benefits the medical profession [110], and allow a better planning and monitoring of hospital activity from a central – rather than regional – level. Other elements of national coherence were reinforced. There is a definition of a national – rather than regional – expenditures target and decisions concerning hospital planning and investment are made at the central – rather than local – level. Despite the differences with Anglo-Saxon reforms, such as the whole-of-government accounts in the UK [111], greater joining-up [112], and agency coordination in New Zealand [113] and Australia [114], French administrative reforms share the same goals: help rebuild the state [115] and reassert the center [116, 117] that lost some of its prerogatives to regions and departments due to decentralization, even if those power gains are at the expense of patients-citizens. Eventually, that recentralization will not only benefit top political and top civil servants, but also a business oligarchy of private clinics, private insurance groups, and consulting firms.

### Conclusion: toward a reconfiguration of the French Welfare State

In theory, NPM reforms will generate efficiencies in the French health care system. However, these were implemented more for pragmatic aims (the need to instill a budget discipline, a 3% deficit under EU regulations, an objective which is shared by both the leftists and the rightists, and to control regions that have a tendency to overspend) rather than for ideological reasons (the conversion to a liberal market economy). Moreover, the French psyche does not welcome the idea of competition within the public sector, and health care services are inherently public rather than private goods in France [118]. Instead, ‘fiscal pragmatism’, top civil servants, and supranational institutions (e.g., EEC, bond rating agencies) demand reforms to balance the budget. These new policies are intended to redeploy the state interventionist powers from ownership of care facilities to hospital governance and to the definition of a national health policy at the expense of policy making and implementation at the local level. The use of DRG accounting and the definition of clear lines of responsibility (a ‘power vertical’ from the Ministry of Health to the hospital managers and Regional Health Agencies) are designed to accelerate reform execution. They do not indicate a withdrawal of the state from public affairs. Greater reliance on market-based mechanisms, reduced state-ownership, and rising private financing [119] imply more – not less – government regulations (e.g., regarding the pricing of health services, establishment of care providers) to manage hospital competition, as in the Anglo-Saxon model [120]. This theory of an active regulatory welfare state [5, 121], which is “*not based on the extension of the public sphere, but on the reduction of the autonomy of non-state actors*”, relies on standards (DRGs, GPs pay-for-performance schemes) and regulations of new entrants, for instance private operators, to govern ‘at a distance’ [122, 123]. Despite these advances, major public issues remain unresolved. These include the shortage of physicians and expensive equipment, such as MRI, and the unavailability of emergency services in many rural areas. With their effectiveness being questioned in France [16, 86, 94] and abroad [124], NPM reforms have stalled (for instance, the objective of leveling up the ‘playing field’ via a convergence of DRG payments for public and private hospitals was delayed until 2018) and, with the arrival of president Hollande in May 2012, priorities shifted to other pressing issues, such as the reintegration of physicians into hospital governance [125], and a focus on quality and responsiveness rather than market-oriented reforms.

## Endnotes

^a^http://www.capital.fr/finances-perso/dossiers/finances-publiques-regions-et-departements-sont-de-plus-en-plus-endettes-692972.

^b^http://www.lemonde.fr/politique/article/2013/11/06/la-decrue-des-partenariats-public-prive-en-france_3508986_823448.html.

## Abbreviations

DRG: Diagnostic-Related Groups
GP: General practitioner
HPST Act: Hospital, Patients, Health, and Territories Act
NPM: New Public Management
PPP: Public-Private Partnerships
RHA: Regional Hospital Agencies
WHO: World Health Organization.
